# Immuno-detection of dioxins using a recombinant protein of aryl hydrocarbon receptor (AhR) fused with *sf*GFP

**DOI:** 10.1186/s12896-016-0282-9

**Published:** 2016-06-21

**Authors:** Walaa Faiad, Abdulsamie Hanano, Mohamed Maher Kabakibi, Abdul Qader Abbady

**Affiliations:** Department of Animal Biology, Faculty of Sciences, Damascus University, Damascus, Syria; Division of Toxicology, Department of Molecular Biology and Biotechnology, Atomic Energy Commission of Syria (AECS), Damascus, Syria; Division of Microbiology and Immunology, Department of Molecular Biology and Biotechnology, Atomic Energy Commission of Syria (AECS), Damascus, Syria

**Keywords:** TCDD, *sf*GFP, AhR, Protein expression, Fusion proteins, Cloning

## Abstract

**Background:**

Dioxins are one of the most toxic groups of persistent organic pollutants. Their bioaccumulation through the food chain constitutes a potential risk for human health. Upon cell entry, dioxins bind specifically and firmly to the aryl hydrocarbon receptor (AhR), leading to the stimulation of several enzymes responsible for its detoxification. Dioxin/AhR interaction could be exploited as an affordable alternative to a variety of analytical methods for detecting dioxin contamination in the environment.

**Results:**

In this work, the ligand binding domain (LBD) of the AhR was cloned downstream a superfolder form of the green fluorescent protein (*sf*GFP), resulting in the construct pRSET-*sf*GFP-AhR. High level of expressed *sf*GFP-AhR fusion protein (50 kDa) was recovered from the inclusion bodies of *E. coli* by simple solubilization with the Arginine, and purified by affinity chromatography via its N-terminal 6 × His tag. Its purity was confirmed by SDS-PAGE analysis and immunoblotting with anti-His or anti-GFP antibodies. Indirect ELISA revealed the ability of the *sf*GFP-AhR, but not the *sf*GFP, to bind to the immobilized dioxin with the possibility to detect such interaction by both its 6 × His and GFP tags,Competitive ELISA showed that anti-dioxin antibody was more sensitive to low dioxin concentrations than *sf*GFP-AhR. Nevertheless,the detection range of *sf*GFP-AhR fusion was much wider and the detection limit was of about 10 ppt (parts per trillion) of free dioxin in the tested artificial samples.

**Conclusions:**

this highly expressed and functional *sf*GFP-AhR fusion protein provides a promising molecular tool for detecting and quantifying different congeners of dioxins.

**Electronic supplementary material:**

The online version of this article (doi:10.1186/s12896-016-0282-9) contains supplementary material, which is available to authorized users.

## Background

Collectively termed as dioxins, polychlorinated dibenzo-p-dioxins (PCDDs) and polychlorinated dibenzofurans (PCDFs) are the most toxic group of the persistent organic pollutants (POPs). Depending on the chlorination level (P = 1–8), dioxins form a generic group of 75 PCDD congeners and 135 PCDF congeners with varying degrees of toxicity. The congeners with chlorine atoms substituted in the lateral 2, 3, 7 and 8 positions of the aromatic rings are considered as the most toxic. 2,3,7,8-Tetrachlorodibenzo-*p*-dioxin (TCDD), with a toxic equivalency factor (TEF) of 1.0, is the most toxic congener of dioxins [1]. Consequently, TCDD was used as a good candidate for investigations of the physiological and toxicological effects of this class of chemicals [2, 3].

Dioxins are a major product of industrial, municipal and domestic incineration and combustion processes [4] and during industrial processes involving chlorinated aromatic and aliphatic compounds, such as pesticides and herbicides synthesis [5]. Currently, incineration of the solid wastes contributes the most to the release of these compounds into the environment [6].

Due to their persistence and high lipophilicity, PCDD/Fs are subject to lipophilic bio-concentration and accumulation in the food chain [7]. Their adverse health effects are now well reported; for example the wasting syndrome [2], immunotoxicity [8], teratogenicity [3], dysfunctional immune and reproductive systems [9] and carcinogenesis [10]. An effective and serious contamination with dioxins has been previously reported in soils, sediments, air and water [11, 12]. In this context, numerous analytical techniques have been developed for the detection and quantification of dioxins. High resolution gas chromatography combined with high resolution mass spectrometry (HRGC/HRMS) is considered the most reliable and sensitive method for separation, identification and determination of the congener-specific concentrations of dioxins [13]. However, this technique requires a sophisticated platform and highly trained personnel, besides being very costly and time-consuming. Consequently several rapid and inexpensive screening assays have been proposed. Immuno-detection, which depends on the ability of a certain compound to be precisely recognized and bound by a specific antibody, makes the base of what is known as the enzyme-linked immunosorbent assay (ELISA) [14, 15]. Alternatively, the detection of signal transduction effects that are related to dioxin stimulation *in vitro* or in cells has been exploited using sophisticated methods; such as the chemical activated luciferase gene expression (CALUX) system [13].

The biological effects of the dioxins in the body critically depend on a cytosolic protein, the aryl hydrocarbon receptor (AhR) [16]. The AhR resembles nuclear receptors (among which it was initially categorized) acting as a ligand-activated transcription factor [16]. In response to activation by dioxins, AhR signalling pathway modifies the expression levels of numerous genes. The best characterized of these pathways at the molecular level is that leading to the induction of the gene for a Phase I drug-metabolizing enzyme; CYP1A1 [17]. In the dormant state, the AhR resides in the cytosol in a protein complex also containing a dimer of heat shock protein 90 (HSP90), an AhR-associated protein-9 and p23 [18]. These chaperone proteins stabilize the AhR maintaining it in a conformation that is unable to enter the nucleus but optimal for ligand binding [19]. The AhR then dissociates from the chaperones and heterodimerizes with the AhR nuclear translocator (ARNT) [20]. AhR/ARNT dimer binds to the major groove of the DNA helix at specific sites [21]. Structurally, the AhR belongs to basic Helix–Loop–Helix/Periodic, AhR nuclear translocator, single-minded (bHLH/PAS) proteins. The AhR contains several important structural elements; including an N-terminal bHLH domain, a C-terminal transcriptional activation domain and a central PAS domain containing two degenerated repeats (PAS-A and PAS-B). The ligand binding domain (LBD) of the AhR is localized between amino acids 230 and 397 [22], and has a similar affinity for TCDD binding as the full-length AhR [23].

In line of these insights we investigated the possibility to fuse the LBD of the AhR with a marker protein, the green fluorescent protein (GFP), in order to exploit the resulting recombinant fusion protein in dioxins detection. The GFP from *Aequorea* jellyfish is widely used as an excellent expression tag for several fusion proteins [24], and it has been expressed in a variety of species; including bacteria, plants, *Drosophila melanogaster, Caenorhabditis elegans,* zebrafish*,* andmammals [24–26]. Waldo and co-workers reported the engineering of an enhanced superfolder (*sf*) form of the GFP, and this *sf*GFP showed increased resistance to denaturation, improved folding kinetics, and increased resistance to aggregation during protein expression [27]. Therefore, the main objective of the current study was to express the *sf*GFP-AhR fusion protein in *E. coli*. Computational 3D structure prediction of this fusion suggested a total accessibility of the AhR moiety for TCDD binding, and consequently this was confirmed in our immunological experiments. Optimized *sf*GFP-AhR fusion could effectively be invested in as an affordable, rapid, sensitive and environment-friendly technique to detect the presence of dioxins in environment samples and food commodities.

## Methods

### Preparation of the AhR cDNA from HepG2 cell line

Total RNA was extracted from the human hepatoma cells (HepG2) using IllustraRNAspin Mini Kit (GE Life Sciences) according to the manufacturer's manual. 2 μg of the RNA was reverse-transcribed to the cDNA using Ready-to-Go You-prime first-strand-beads (GE Life Sciences) with oligo-dT^15–18^ (Invitrogen). 2 μl of the cDNA was used as a template in a PCR with a pair of specific primers for the AhR(LBD); AhR(*Bam*HI)-F and AhR(*Eco*RI)-R (Additional file 1: Table S1). Primers were designed to amplify the LBD of the *AhR* gene and to add *Bam*HI and *EcoR*I restriction sites at the 5' and 3' ends, respectively. The fragment of LBD was amplified by a high fidelity Taq DNA polymerase (AccuPrime™ Kit; Invitrogen) at 55 °C annealing temperature. Amplified AhR fragment and the pRSET-*sf*GFP plasmid [28] were digested with *Bam*HI and *Eco*RI (Fermentas) then ligated using the T4 DNA ligase following standard procedures (Fermentas).

### Bacterial strains and growth conditions

*E. coli* strains TOP10 (Invitrogen) and BL21(DE3) Gold (Novagen) were used in cloning and protein expression. *E. coli* was grown in Luria Broth (LB; 1 % Tryptone, 0.5 % yeast extract, 171 mM NaCl) (Bio Basic Inc.) with ampicillin antibiotic (100 μg/ml, Sigma) in orbit-rotating incubator at 37 °C. Freshly prepared electro-competent *E. coli* TOP10 cells were transformed with the plasmid constructs by electroporation. Colony PCR screening for positive AhR clones was performed using pRSET specific primers (T_7_F/T_7_R) (Additional file 1: Table S1). The plasmid constructs were isolated from some positive clones by plasmid Miniprep Kit (Qiagen) after being grown in LB/ampicillin medium. The identity of these plasmid constructs was confirmed by digestion with the restriction enzymes and by sequencing using different sequencing primers covering the full-length LBD (Additional file 1: Table S1).

### Expression of the sfGFP-AhR fusion protein as inclusion bodies

The confirmed plasmid constructs; the pRSET-*sf*GFP and pRSET-*sf*GFP-AhR, were used to transform *E. coli* BL21 (DE3) Gold cells by electroporation. Protein expression was performed in 250 ml shake flasks by growing the bacteria in LB medium till an optical density of 0.5 to 0.7 was reached, and then the expression was induced with 0.5 mMIsopropylthio-D- galactoside(IPTG, Promega) for 16 h at 15 °C. After centrifugation, the pellet of bacteria was resuspended on the binding buffer(50 mMtris-HCl pH 8.0, 150 mM NaCl and 1 mMphenylmethylsulfonyl fluoride (PMSF, Sigma) supplemented with a complete protease inhibitor cocktail (Roche), then lysed by 8 pulses (15 s each) of sonication at 22 % amplitude (750-Watt Ultrasonic Homogenizer, Cole-Parmer) in ice. The lysate containing soluble proteins as the *sf*GFP was cleared by centrifugation at 12000 × g for 20 min at 4 °C. The pellet of the inclusion bodies containing insoluble proteins (as the *sf*GFP-AhR) was re-suspended by sonication in the same binding buffer supplemented with (5 mM DTT, 5 mM EDTA, 1 M urea and 1 % triton-x). This washing step is designed to remove contaminants, especially proteins that may have adsorbed onto the hydrophobic inclusion bodies during processing and could affect protein refolding yield. After washing with the basic binding buffer to eliminate the detergents, inclusion bodies were ready to be solubilized using the different reagents.

### Refolding and solubilizion of *sf*GFP-AhR inclusion bodies

To assess the optimal reagent for assisting the correct refolding of the *sf*GFP-AhR, inclusion bodies were prepared at 0.1 mg/ml TBS buffer (20 mMtris-HCl pH 8.0 and 150 mM NaCl). 500 μl of this preparation was distributed into several 1.5 ml microtubes then centrifuged at max speed for 5 min. The pellets in the microtubes were dispersed and dissolved by sonication in the same buffer containing different concentration of various additives reported in the literature [29] (Additional file 2: Table S2). After 30 min of incubation at RT, supernatant (soluble fraction) was recovered from each condition by centrifugation and the remaining pellet of insoluble *sf*GFP-AhR was dissolved by sonication in 500 μl of 8 M urea-containing buffer (insoluble fraction). The formation of a fluorophore in the soluble and insoluble fractions was monitored by measuring the fluorescence density, expressed as a relative fluorescent unit (RFU), at excitation wavelength 485 nm with emission wavelength 538 nm (Fluoroskan Ascent, Thermo Labsystems), and the fluorophore fluorescein (Sigma) was used as a control.

### Purification of the *sf*GFP and *sf*GFP-AhR fusion proteins

Soluble fractions of *E. coli* extract containing the *sf*GFP and Arginine-refolded *sf*GFP-AhR were dialysed in binding buffer containing 20 mM imidazole and then purified using a 5 ml column of nickel charged nitrilotriacetic acid (NTA) superflow sepharose (Qiagen) installed on the fast protein liquid chromatography (FPLC) AKTA prime plus system (GE Life Sciences). After washing, bound proteins were eluted from the column with 500 mM imidazole-containing buffer. The eluted fraction was concentrated on Vivaspin concentrators with a molecular mass cut-off of 10 kDa (Vivascience). The concentration of the purified proteins was determined by the Bradford method, and their purity was evaluated in SDS-PAGE prepared using stacking gel 5 % and running gel 12 %. After electrophoresis, the gel was stained in coomassie brilliant blue buffer (45 % methanol, 10 % acetic acid and 0.25 % coomassie R250) for 2 h and then washed several times in the destaining buffer.

### Immunoblotting of the sfGFP and sfGFP-AhR

Purified proteins were separated by SDS-PAGE before blotting onto 0.45 μm nitrocellulose membranes (Bio-Rad) using 1× transfer buffer (25 mMtris-HCl, 200 mM glycine, 0.1 % SDS and 20 % methanol). After blotting, the membranes were washed with TBS-T (0.05 % Tween-20 in TBS) then incubated in the blocking buffer (5 % skimmed milk in TBS-T). The membranes were incubated with 1:2000 dilution of either mouse anti-GFP (Roche) or mouse anti-6 × His (R&D Systems) antibodies for 1 h at RT. After several washes with TBS-T, the membranes were incubated with (1:2000) dilution of a secondary goat anti-mouse antibody conjugated to the horseradish peroxidase (HRP, Bethyl laboratories) for 1 h at RT. Bands revelation was achieved by adding 3-amino-9-ethylcarbazole (AEC, Sigma) chromogen substrate in acetate buffer in the presence of a hydrogen peroxide.

### Indirect ELISA assay for testing sfGFP-AhR

An indirect ELISA was performed by immobilizing the antigens (10 nM), *sf*GFP-AhR or *sf*GFP, on a standard MaxiSorp 96-well plate (Nunc) overnight in a bicarbonate/carbonate coating buffer (100 mM, pH 9.6). Alternatively, dioxin binding was tested using a TCDD-pre-coated 96-well ELISA microplate (Abraxis LLC). Before use, all different plates were incubated in the blocking buffer (5 % skimmed milk in TBS-T) for 2 h at RT then washed 3 times with TBS-T. Immobilized TCDD in the wells was directly detected using a rabbit-anti-TCDD (Abraxis), following the manufacturer's instructions. Otherwise, the *sf*GFP-AhR and *sf*GFP were diluted to the desired concentration (100 nM) in 1 % blocking buffer then applied to the wells (100 μl) for 16 h at 4 °C, then washed 3 times with TBS-T. Antigens (immobilized or bound to TCDD) were detected by an incubation for 1 h at RT with a diluted (1:2000) mouse anti-6 × His, rabbit anti-6 × His (Bethyl laboratories), mouse anti-GFP or rabbit anti-GFP (a homemade product, [28]). After 5 washes, diluted (1:2000) goat anti-mouse or goat-anti-rabbit IgGs conjugated to horseradish peroxidase (HRP, Bethyl laboratories) were added to the appropriate wells. The wells were then washed 5 times before bound conjugates were detected with 50 μl of 3,3′,5,5′-tetramethylbenzidine (TMB, Sigma) ready-to-use substrate. The reaction was finally stopped with the addition of 50 μl of 1 M H_2_SO_4_. The spectroscopic absorbance of each well was measured in an automated plate reader at a wavelength of 450 nm.

### TCDD detection by a competitive indirect ELISA using the *sf*GFP-AhR

Using the same TCDD-pre-coated plates and doing the same primary steps of blocking and washing, the competitive ELISA was carried out by incubating the *sf*GFP-AhR (30 nM) or rabbit-anti-TCDD with serial dilutions (from 0 – to 100,000 parts per trillion (ppt) in 1 % DMSO-containing blocking buffer) of free TCDD (stock at 10 μg/ml, purity 99 %, Supelco Inc.) in 100 μl volume for 2 h at RT. Different conditions were then transferred to TCDD-pre-coated wells and incubated for further 2 h at RT. The following steps were performed as described earlier using a rabbit-anti-GFP antibody, goat-anti-rabbit-HRP and the TMB substrate. The experiments were performed three times to determine the mean values (used to draw the graphs) and the error bars were calculated by dividing the standard deviation of the data by the square root of number (3) of values that make up the mean.

## Results

### Design of the *sf*GFP-AhR fusion protein

**Fig. 1:**
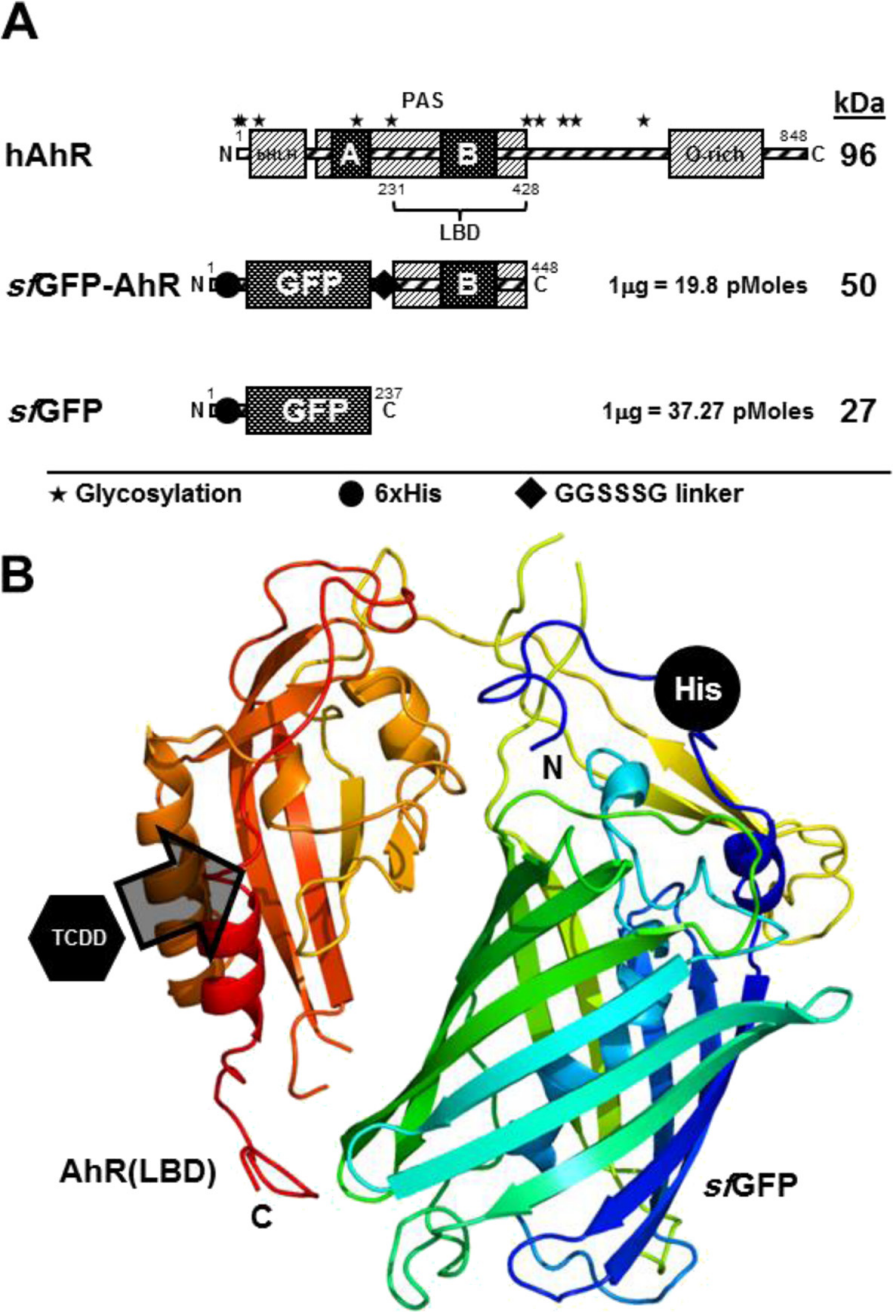
Designing of the *sf*GFP-AhR fusion protein. **a** Schematic representation of the hAhR gene and the two recombinant proteins; sfGFP-AhR and sfGFP, used in this study. The different domains of the AhR gene are shown and the cloned LBD domain (231–428 aa) is indicated. The theoretical molecular size (kDa) and molecular weight (pMoles/μg) are shown to the right of each recombinant construct. Positions of the different elements; 6 × His tag, GGSSSG linker and glycosylation sites, are indicated using specific symbols ●, ♦ and *, respectively. **b** Cartoon representation of the modelled 3D structure of the sfGFP-AhR fusion, where TCDD binding cavity and the N-terminal 6 × His tag are shown. Structure simulation was predicted using Phyre2 server [47]

Human AhR is a relatively large protein of 96 kDa composed of 848 amino acids (aa), extending over several well identified functional domains (Fig. 1a). LBD positioned from 231 to 428 was cloned downstream of the *sf*GFP gene in the plasmid pRSET-*sf*GFP, resulting in a fusion protein of 50 kDa (Fig. 1a). The resulting *sf*GFP-AhR fusion protein contains an N-terminal 6 × His-tag and a free and flexible LBD moiety exposed an internal polypeptide linker GGSSSG at its C-terminal (Fig. 1a). A soluble cytoplasmic N-terminal 6 × His-tagged *sf*GFP of 27 kDa was also expressed in *E. coli* using the original plasmid pRSET-*sf*GFP (Fig. 1a) [28, 30]. A simple 3D-modeling of the cloned LBD showed the cavity for TCDD binding that extends from 284 to 390 aa, overlapping entirely with the PAS-B domain and containing several aa whose side chains are critical for TCDD binding. These aa are found to be highly conserved between human, mouse and rat as well as many other mammalian species [31], and the main secondary structures (the alpa-helixes and the beta-chain strands) of the cavity are also conserved (Additional file 3: Figure S1). As inferred from the 3D structure prediction of the fusion protein, this cavity appeared to be exposed away from the *sf*GFP and in a favourable orientation for TCDD binding (Fig. 1b, Additional file 1: Table S1).

### Cloning of *AhR* gene into the pRSET-*sf*GFP plasmid

**Fig. 2:**
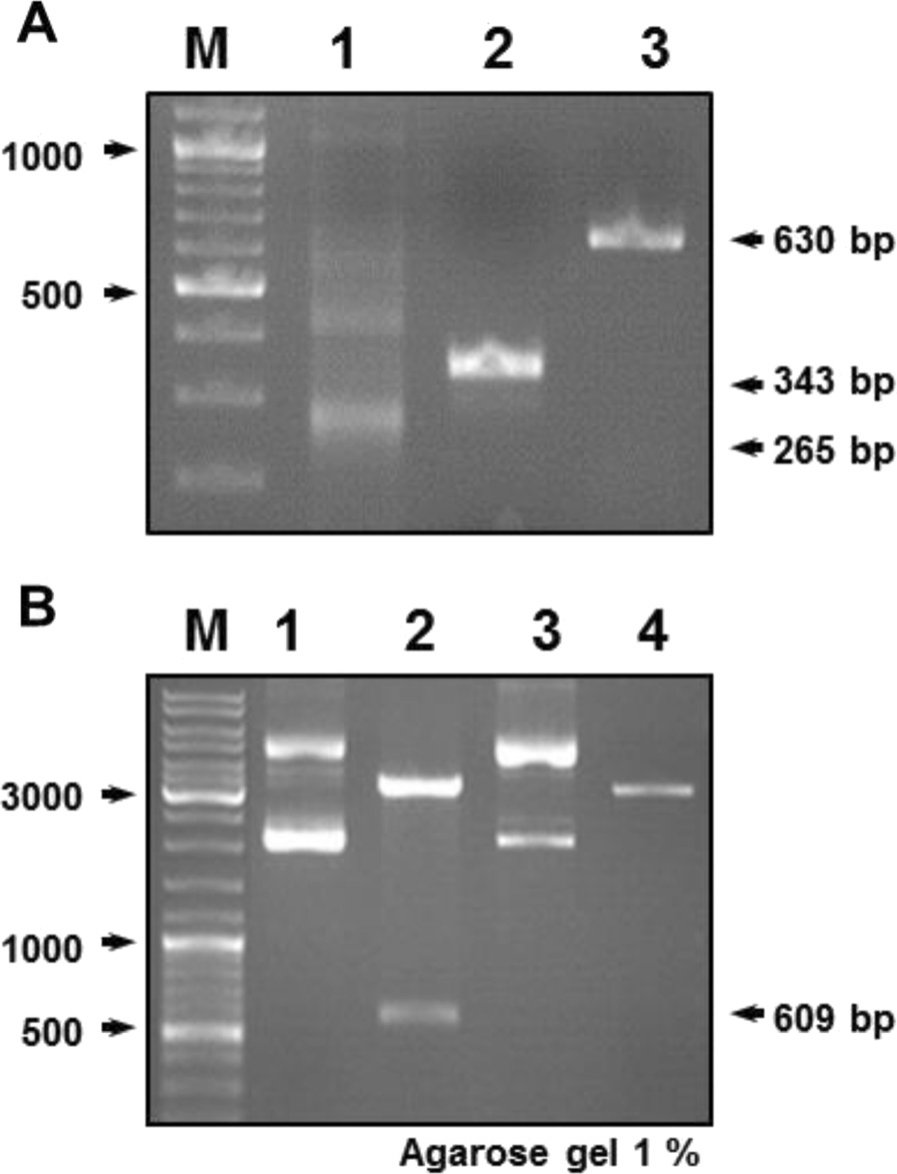
Construction of the pRSET-*sf*GFP-AhR plasmid. **a** PCR amplification was performed on the cDNA of HepG2 cells and the products were separated on a 1 % agarose gel; a fragment (265 bp) of *actin* gene as a control (lane 1), small domain from the *AhR* gene using internal primers (lane 2) and the full-length of LBD (lane 3). Expected sizes of the amplified bands are shown to the right. **b** 1 % agarose gel containing the pRSET-*sf*GFP-AhR (lanes 1&2) and pRSET-*sf*GFP (lanes 3&4); undigested (lanes 1&3) or digested with *Bam*HI/*Eco*RI restriction enzymes (lanes 2&4). The extracted AhR insert is indicated with an arrow to the right

The AhR(LBD) was amplified from the cDNA of the HepG2 cell line using a couple of AhR-specific primers, and a single DNA fragment of 630 bp was therefore obtained and cloned in the pRSET-*sf*GFP plasmid (Fig. 2a). After transformation in *E. coli*, positive colonies were selected and used for plasmid mini-preparation and the correct constructs were verified by *Bam*Hl/*Eco*RI-digestion (Fig. 2b). An expected band of 609 bp corresponding to the LBD fragment was only resulted from digesting the construct pRSET-*sf*GFP-AhR and not the original plasmid (Fig. 2b). Furthermore, the cloned insert in the pRSET-*sf*GFP-AhR was entirely sequenced and aligned to the human *AhR* gene sequence (Accession: NM_001621) to confirm the absence of mutations.

Plasmids like the pRSET with a T_7_ promoter are widely used for high throughput protein expression in *E. coli* strains carrying the lambda DE3 lysogen [32] (Additional file 4: Figure S2 and Additional file 2: Table S2).

### Expression of the fluorescent *sf*GFP-AhR fusion protein

**Fig. 3:**
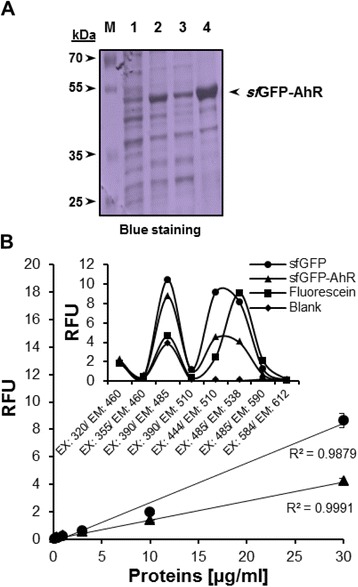
Expression of the fluorescent *sf*GFP-AhR. **a** SDS-PAGE (acrylamide 12 %) separation of protein samples obtained after the expression of the *sf*GFP-AhR, showing total cell extract before (lane 1) and after 16 h (lane 2) of IPTG induction, soluble fraction (lane 3) and the lysate of the inclusion bodies in 8 M urea (lane 4). Detection of migrated proteins was done by a coomassie blue staining. **b** Fluorescence of serial concentrations of the *sf*GFP and *sf*GFP-AhR was measured at the wavelength 485 nm for excitation (EX) and 538 nm for emission (EM). The values were expressed as a relative fluorescent unit (RFU) and the accuracy is shown next to each curve (R^2^). (Inset) Fluorescence spectra of the different proteins (30 μg/ml) and the fluorophore fluorescein (1 μg/ml) were determined by measuring RFU at available pairs of wavelengths on the Fluoroskan Ascent® microplate reader. Blank conditions represent the fluorescence of PBS-containing wells

Expression of the *sf*GFP-AhR was carried out after transformation of *E. coli* BL21 (DE3) Gold cells with the confirmed pRSET-*sf*GFP-AhR construct. A remarkable expression of a 50 kDa green *sf*GFP-AhR fusion protein was observed after IPTG induction. Unexpectedly, the expressed *sf*GFP-AhR was mainly accumulated in the form of the inclusion bodies, and a small amount of the protein was foundsoluble in the cytoplasm (Fig. 3a). Fluorescence properties of the *sf*GFP-AhR fusion protein, recovered from the inclusion bodies by solubilizing in urea, were investigated using serial concentrations. Apparently, the *sf*GFP-AhR exhibited a fluorescence, but to a lesser extent than the *sf*GFP, at a standard wavelength for excitation (485 nm) and emission (538 nm) (Fig. 3b). However, fluorescence spectra of the free and fusion *sf*GFP seemed to be identical at several pairs of wavelengths for excitation and emission and differ slightly from the spectrum of the fluorescein (Fig. 3b, inset).

### Optimization of *in vitrosf*GFP-AhR refolding

**Fig. 4:**
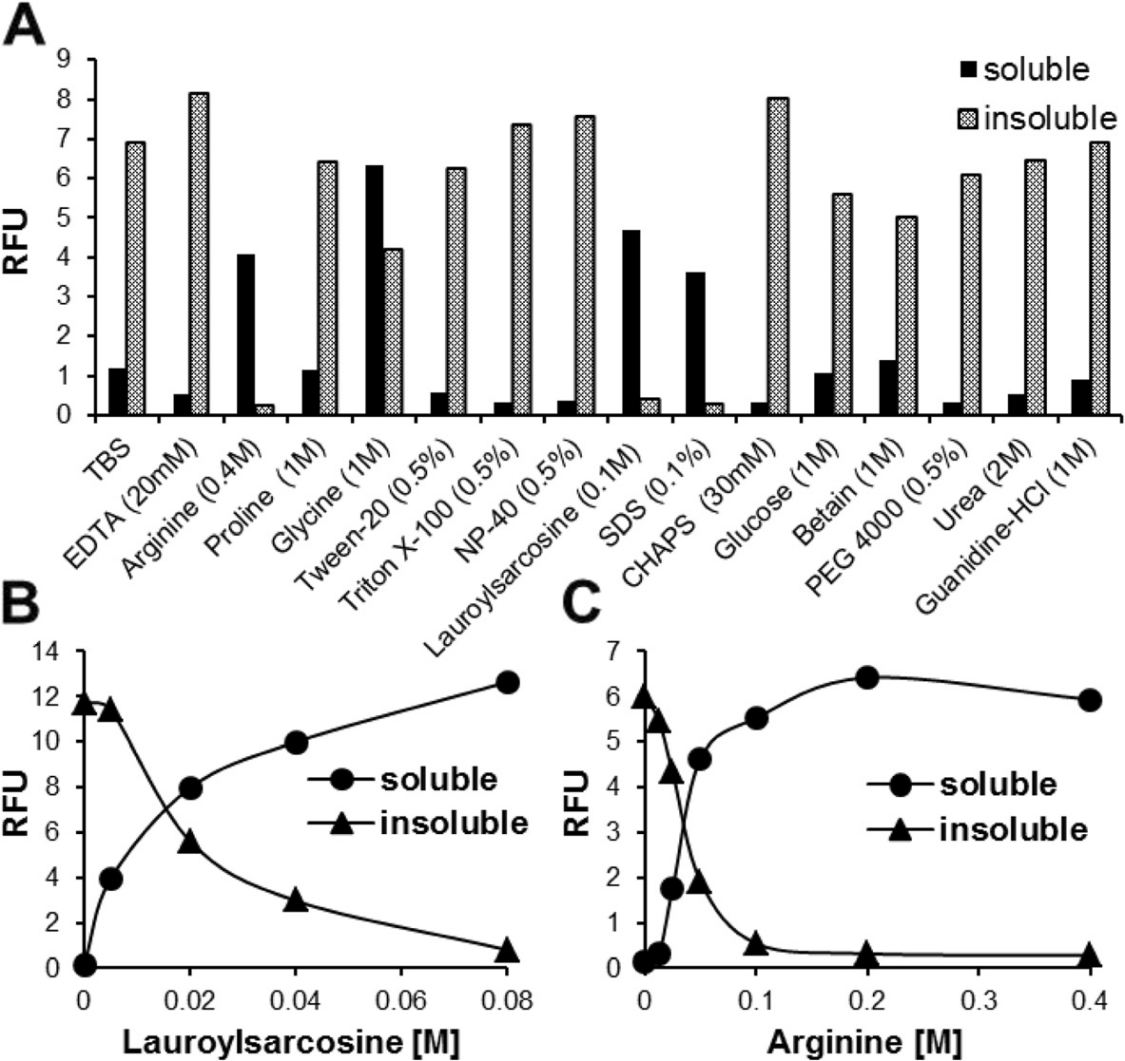
Optimization of the *in-vitro sf*GFP-AhR refolding. **a** Several additives were added at their indicated optimal concentration to solubilize a pellet (0.1 mg in 1 ml) of *sf*GFP-AhR inclusion bodies (soluble fraction). Remaining insoluble precipitates were recovered by centrifugation and solubilized in 8 M urea (insoluble fraction). Serial molar concentrations of Lauroylsarcosine (**b**) and Arginine (**c**) used for solubilizing the pellet of *sf*GFP-AhR inclusion bodies. The RFU was measured (EX: 485/EM: 538) in soluble and insoluble fractions for each additive and for the different concentrations of the last two additives

Due to the insolubility of the expressed AhR-*sf*GFP fusion, an *in vitro* solubilizing and refolding process was required to obtain an active form of the protein. Several reagents were added into the buffer of the inclusion bodies to improve the solubility and prevent the aggregation of the *sf*GFP-AhR protein as described in Materials and Methods. By comparing the fluorescence in soluble and insoluble fractions, only three reagents (0.4 M Arginine, 0.1 M Lauroylsarcosine and 0.1 % SDS) were able to to solubilize the inclusion bodies completely, and no remaining fluorescence could be detected in the insoluble fraction (Fig. 4a). Other used reagents were not as effective as these three conditions in solubilizing the *sf*GFP-AhR. Next, serial concentrations of Arginine (from 0 to 0.4 M) and Lauroylsarcosine (from 0 to 0.08 M) were examined for optimizing the solubilization of *sf*GFP-AhR inclusion bodies. As we did before, the fluorescence of the *sf*GFP-AhR was measured in soluble and insoluble fractions. Effectively, protein solubility was enhanced using increasing concentrations of both reagents (Fig. 4b and c). Consequently, 0.1 M Arginine was used for refolding and solubilizing of the *sf*GFP-AhR fusion from the inclusion bodies.

### Purification and immunodetection of the *sf*GFP-AhR

**Fig. 5:**
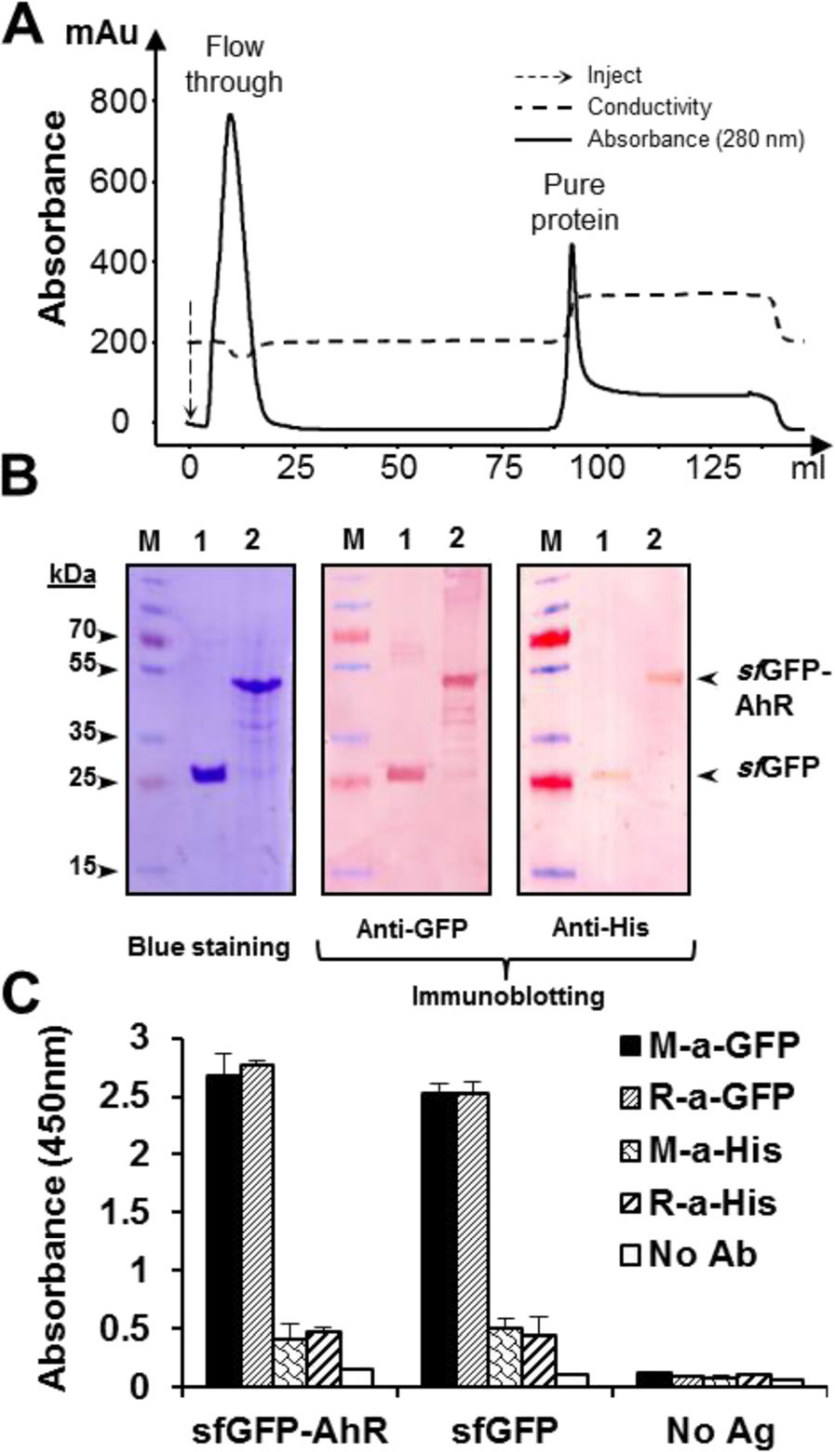
Purification and characterization of the *sf*GFP-AhR. **a** Diagram of the purification procedure using Ni^+^-NTA column installed on FPLC AKTA prime system. Continuous line represents the absorbance of the eluate, and the peaks of the flow-through sample and purified protein (*sf*GFP-AhR) are indicated. Dashed line represents conductivity of the eluate. **b** Detection of the purified *sf*GFP and *sf*GFP-AhR was done after SDS-PAGE separation either by blue staining or by immune blotting using anti-GFP or anti-6 × His antibodies. The protein molecular weight ladder is in the first lane (M). **c** Indirect ELISA for testing the purified *sf*GFP-AhR and *sf*GFP that were immobilized (10nM) on the microplate and detected by polyclonal and monoclonal anti-GFP or anti-6 × His tag antibodies (1:2000)

Purification of the *sf*GFP-AhR from the solubilized inclusion bodies was performed on an immobilized-metal affinity chromatography using Nickel-charged NTA column (Fig. 5a). The integrity of the purified proteins, the *sf*GFP-AhR and *sf*GFP, was analysed by SDS-PAGE and immunoblotting by either mouse anti-GFP or anti-6 × His antibodies. Two major specific bands of about 50 and 27 kDa, corresponding to the *sf*GFP-AhR and *sf*GFP, respectively, were immune-detected on the membranes (Fig. 5b). The purified *sf*GFP-AhR and *sf*GFP were tested in a solid-phase ELISA through targeting their GFP and 6 × His tags using different monoclonal and polyclonal antibodies. Obviously, the GFP tag allowed much more efficient detection of both ELISA-immobilized proteins compared to the 6 × His tag, and this could be related to the great differences in the size and epitopes availability of these two tags (Fig. 5c). These data indicated clearly that the immunodetection of the *sf*GFP-AhR fusion could be achieved efficiently using specific anti-GFP antibodies.

### Detection of the interaction of the *sf*GFP-AhR with dioxin

**Fig. 6:**
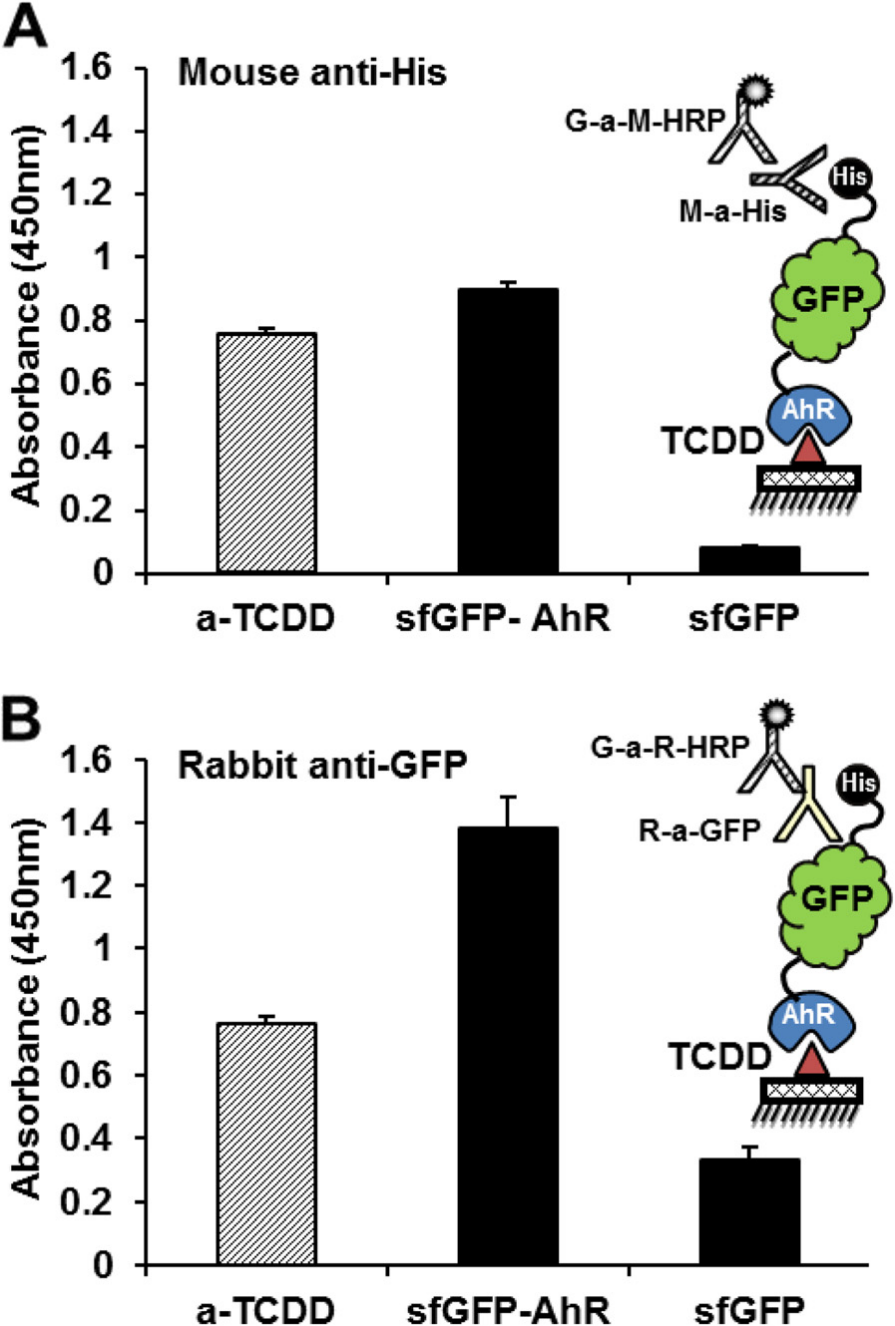
Testing the interaction of the *sf*GFP-AhR with the TCDD by ELISA Indirect ELISA was performed using TCDD-pre-coated microplate. Anti-TCDD, sfGFP-AhR (100 nM) and sfGFP (100 nM) were added to the wells and the interaction was detected either by an anti-6 × His (**a**) or anti-GFP (**b**) antibodies. The graphical inset in each panel explains the principle of the detection method

**Fig. 7:**
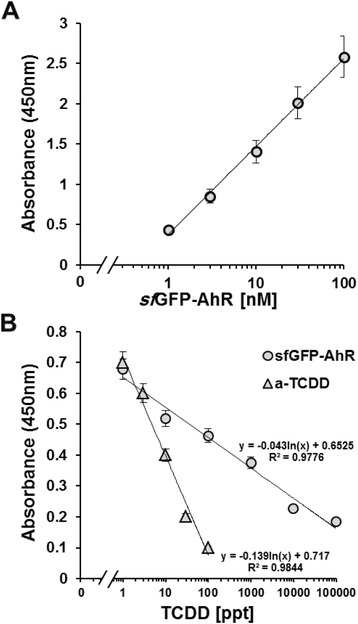
TCDD detection by the *sf*GFP-AhR-based competitive ELISA. **a** Optimal concentration of the *sf*GFP-AhR for the assay was determined by indirect ELISA using serial logarithmic concentrations (nM) for incubation in TCDD-pre-coated wells. **b** Competitive ELISA was performed on serial logarithmic concentrations (ppt) of free TCDD, which were incubated with the rabbit-a-TCDD or the *sf*GFP-AhR (30 nM) before being transferred into the TCDD-pre-coated wells. The detection of bound *sf*GFP-AhR in both types of ELISA was performed using a rabbit anti-GFP, and both rabbit sera were detected with a goat anti-rabbit-HRP. The logarithmic fit equation and the accuracy (R^2^) are shown next to each curve

The *sf*GFP-AhR was effectively able to detect the immobilized TCDD using either anti-6 × His or anti-GFP antibodies (Fig. 6a and b). However, the major absorbance at 450 nm (about 1.4) was detected in the case of anti-GFP, and the *sf*GFP was used as a negative control (Fig. 6b). As a positive control, the immobilized TCDD gave an absorbance of 0.8 when detected using specific anti-TCDD (a-TCDD) antibody. Competitive ELISA is usually used as an analytical method to quantify dioxins in food, feed and environmental soil samples. The principle of the competitive ELISA is that free dioxins in the samples compete with the immobilized dioxin for binding to the detecting molecule (*sf*GFP-AhR or a-TCDD). Therefore, we used this system of detection to confirm the sensitivity of the *sf*GFP-AhR fusion towards TCDD. For this purpose, we firstly determined the optimal effective concentration of the *sf*GFP-AhR necessary for achieving 75 % of the maximal detection of the immobilized TCDD (EC^75^ = nM). To do that, serial logarithmic molar concentrations of the *sf*GFP-AhR were incubated in the wells of TCDD-pre-coated microplate then the binding activity was evaluated using a rabbit-anti-GFP antibody. A linear correlation between the absorbance and the concentrations of the *sf*GFP-AhR was clearly observed, and EC^75^ of the detection was estimated to be about 33.1 ± 9.2 nM (Fig. 7a). Based on this data, a *sf*GFP-AHR concentration of 30 nM was subsequently used. Different concentrations of TCDD (in ppt) were incubated with either the *sf*GFP-AhR or a-TCDD, and then added into the wells of the TCDD-pre-coated microplate. Binding of the detecting molecule to immobilized TCDD was finally revealed by the secondary antibodies. Absorbance values were inversely proportioned to the amount of free TCDD in the samples. In the case ofthea-TCDD, EC^50^ of the detection was 11.9 ± 0.4 ppt and the detection range (from EC^99^ to EC^1^) was from 1.1 ± 0.05 ppt to 83.7 ± 2.6 ppt, whereas the *sf*GFP-AhR showed a higher EC^50^ of about 346.5 ± 21.4 ppt with a wider detection range from 0.8 ± 0.04 ppt to 51600 ± 15500 ppt (Fig. 7b). In conclusion, the sum of dioxin in the target samples could be detected using a similar method and its exact concentration could be calculated by a comparison with the same standard curve and logarithmic fit equation.

## Discussion

To date, many reports have described the heterologous expression of the recombinant AhR in both prokaryotic and eukaryotic cell types. Particularly, the recombinant expression of the ligand-binding competent AhR would have significant advantages for many bioanalytical applications regarding dioxins detection. The majority of the described methods take advantage of the ability of dioxin/AhR complexes to trigger certain signal transduction pathways [33]. Transgenic tobacco plants carrying the recombinant mouse AhR-mediated β-glucuronidase (GUS) reporter gene expression system was designed and showed a significantly increased GUS activity when treated with certain AhR ligands [34]. Such assays did not need any extraction and purification of chemicals and seemed to be useful for a biochemical assay of dioxins and dioxin-like compounds [35]. Furthermore, recombinant yeast cells *Saccharomyces cerevisiae* have been designed as a dioxin bioassay system, in which the expressed full length human or mouse AhR/Arnt complexes form after ligand binding and interact with a responsive element located on the promoter of ß-galactosidase reporter gene [36], and this system was utilized to identify potent endogenous AhR ligands from human urine and bovine serum [37]. Baculovirus expression vector system was utilized for high level production of the full-length human and rat AhR in *Spodopterafrugiperda* (Sf9), and the recombinant proteins were active in the quantitative analysis of the ligand [38]. In fact, the absence of the disulfide bridges and glycosylation sites in the LBD of AhR makes it a more suitable target for expression in *E. coli*. However, several attempts for LBD expression in *E. coli* were unsuccessful because of its instability and its high tendency to aggregate because of misfolding or incomplete folding processes [23]. Therefore, fusion protein technology is considered one of the best solutions to enhance the protein expression and solubility. For example, it chaperones the proper folding, facilitates the purification and reduces the protein degradation or toxic effects [39]. Many fusion moieties are available to improve the expression and purification of proteins, including glutathione S-transferase (GST), maltose-binding protein (MBP), thioredoxin A (TrxA), and the GFP [40].

In the current study, we successfully produced the LBD of AhR in a fusion with the *sf*GFP, resulting in its expression at a high level (~150 mg/L of bacteria culture) in the cytoplasm of *E. coli*. Despite its well documented stability and solubility, the enhanced form of the GFP, the superfolder GFP, failed to maintain the correct folding of the expressed AhR, leading therefore to its accumulation into the inclusion bodies. This was disappointing since many reports described the recovery of several insoluble proteins from the inclusion bodies simply by their merging with the *sf*GFP, e.g. the human growth hormone [41], and in certain cases, this resulted in the enhancement of their physiological roles [42]. The culture and induction conditions, including temperatures and IPTG concentrations, may play a critical role in the solubility of many recombinant proteins, especially, when some stringent culture conditions provoke the production of large quantities of the exogenous protein that the cell folding machinery fails to process [43]. Hence, several conditions were tested to assess their influence on the solubility of the *sf*GFP-AhR which was simply monitored by comparing the colour of the bacterial lysate and precipitate. Unfortunately, the fusion greenish protein was always found in the inclusion bodies regardless of the conditions used, even with the mild ones; Tm 10 °C and 0.1 mM IPTG, on the contrary, free *sf*GFP was always soluble when expressed from the plasmid pRSET-*sf*GFP, using many different culture conditions (data not shown).

Arginine has long been used as an interesting additive for solubilizing the inclusion bodies and enhancing protein solubility and stability [44]. Its mechanism of action relies on inhibiting the aggregation of misfolded proteins and surrounding their exposed hydrophobic surfaces. Furthermore, 0.5–2MArginine, can be used to actively extract the folded proteins from insoluble pellets obtained after lysing *E. coli* cells [44]. Comparing several tested solubilizing reagents reported in the literature [29], we found that low concentrations of Arginine, e.g. 0.1 M, were enough to refold totally *sf*GFP-AhR from the inclusion bodies, and even without a prior step for solubilizing the inclusion bodies in 6 M guanidine-HCl or 8 M urea. Lauroylsarcosine showed a similar effect on the inclusion bodies of the *sf*GFP-AhR, however its severity, as a strong ionic detergent, compared to the Arginine which is barely a stabilizing amino acid, makes its uses unfavourable in the production procedure of the *sf*GFP-AhR. Nevertheless, we speculated from this experiment that the structure of the *sf*GFP-AhR is moderately misfolded, making its recovery from the inclusion bodies an easy and inexpensive task to perform. This could be related to the *sf*GFP owing to the folding reputation it has, beside, its visible and florescent signals which made it possible to track the transformation of the fusion protein between the insoluble and soluble states, especially when different solubilizing additives were used. Alternatively, the aggregation problem of the AhR can be probably overcome by using eukaryotic heterologous expression systems such as animal or plant cells.

The ability of*sf*GFP-AhR to bind to TCDD was proven by a standard and a competitive ELISA. This last type of ELISA is mainly used for the detection of very-small antigens that are difficult to be immobilized on a solid support, hard to purify and composed of a few number of epitopes, e.g. the haptens [14]. It is usually performed to quantify TCDD contamination in water, soil and sediment samples [12]. Despite the fact that anti-TCDD antibody showed more sensitivity for TCDD in competitive ELISA than the *sf*GFP-AhR, the detection range of the *sf*GFP-AhR was much wider. According to the USA-Environmental Protection Administration (EPA), the maximum limits for dioxins in wastewater are set to be 5 pg I-TEQ per liter for new companies and 10 pg I-TEQ per liter for existing companies. These ranges which equal 5 to 10 ppt of total dioxins are being covered by estimated sensitivity range of the proposed *sf*GFP-AhR detection system. For enhancing the affinity, replacing the LBD in the *sf*GFP-AhR fusion, originated from human AhR, with the equivalent domain from mouse or rat AhR can be suggested, because they are known to have better binding kinetics towards TCDD [38]. Nevertheless, this *sf*GFP-AhR-based immunoassay can be exploited as a method for analyzing large number of samples, from different sources, for contamination with TCDD, or any similar compounds. An estimated value of TCDD content can be attributed to the measured samples simply by comparing the logarithmic curve fit of the known concentrations of standard TCDD. Samples that are determined to be positive in this immunoassay can undergo congener-specific analyses by HR-GC/MS. Beside its analytical uses, the fluorescent *sf*GFP-AhR fusion can be used as a powerful method for bio-tracing of TCDD within the cells or tissues in diverse unexploited areas of research. For example, this bioanalytical method will surely contribute to a better understanding of the plant/dioxin relationship and therefore in deciphering the mechanisms involved in plant responses to dioxin exposure [45, 46].

## Conclusions

In conclusion, dioxin binding domain of the AhR was produced in a fusion with an enhanced form of the GFP using an efficient *E. coli* protein expression system. After Arginine-refolding and affinity purification, the *sf*GFP-AhR fusion protein was able to bind to TCDD using a standard and competitive ELISA, and the detection limit of free TCDD in samples could reach very low levels (~10 ppt).

## Abbreviations

AhR, aryl hydrocarbon receptor; bHLH/PAS, basic helix-loop-helix/Per-ARNT-Sim; GFP, superfolder green fluorescent protein; LBD, ligand binding domain; PCDDs, polychlorinated dibenzo-p-dioxins; PCDFs, polychlorinated dibenzofurans; TCDD, 2,3,7,8-Tetrachlorodibenzo-*p*-dioxin *sf.*

## Additional files

Additional file 1: Table S1. The primers used for the amplification and cloning of the AhR. The different parameters (application, name, length and sequence) of the primers used for *AhR* gene amplification and cloning into the pRSET-*sfGFP* plasmid. (DOC 79 kb) Additional file 2: Table S2. The different additives used for refolding the *sf*GFP-AhR. The basic information of the different additives used for solubilizing the *sf*GFP-AhR from the inclusion bodies. (DOC 95 kb) Additional file 3: Figure S1. Alignment of the AhR(LBD) amino acids sequences. Amino acids sequence alignment of the AhR(LBD) from human (hAhR, NP_001612), mouse (mAhR, NP_038492) and rat (rAhR, NP_037281). Similarity bar above the sequences and the different secondary structures (as attributed by Geneious software) are indicated with small arrows. The TCDD binding cavity and the PAS-B domains are indicated through which small boxes under the sequence refer to the amino acids whose side chains are implicated in the structure of the cavity (light coloured for lateral and dark for internal positions), as described before [31]. (DOC 272 kb) Additional file 4: Figure S2. The structure of the plasmid pRSET-*sf*GFP-AhR. Map of the plasmid construct pRSET-*sf*GFP-AhR, in which the inserted AhR (LBD) is indicated. The most important elements of the plasmid are shown, including the T_7_ promoter, N-terminal 6×His tag, two restriction sites (*Bam*HI/*Eco*RI) used for insert ligation, ampicillin resistance gene (Amp), f1/PUC origin of replication and the *sf*GFP gene. (DOC 266 kb)
