# Supplementary material for: Immuno-detection of dioxins using a recombinant protein of aryl hydrocarbon receptor (AhR) fused with sfGFP
Source: BMC Biotechnol. 2016 Jun 21;16:51. doi: 10.1186/s12896-016-0282-9 (PMC4915173; doi:10.1186/s12896-016-0282-9)

Additional file 1: Table S1 The primers used for the amplification and cloning of the AhR. The different parameters (application, name, length and sequence) of the primers used for *AhR* gene amplification and cloning into the pRSET-*sfGFP* plasmid.


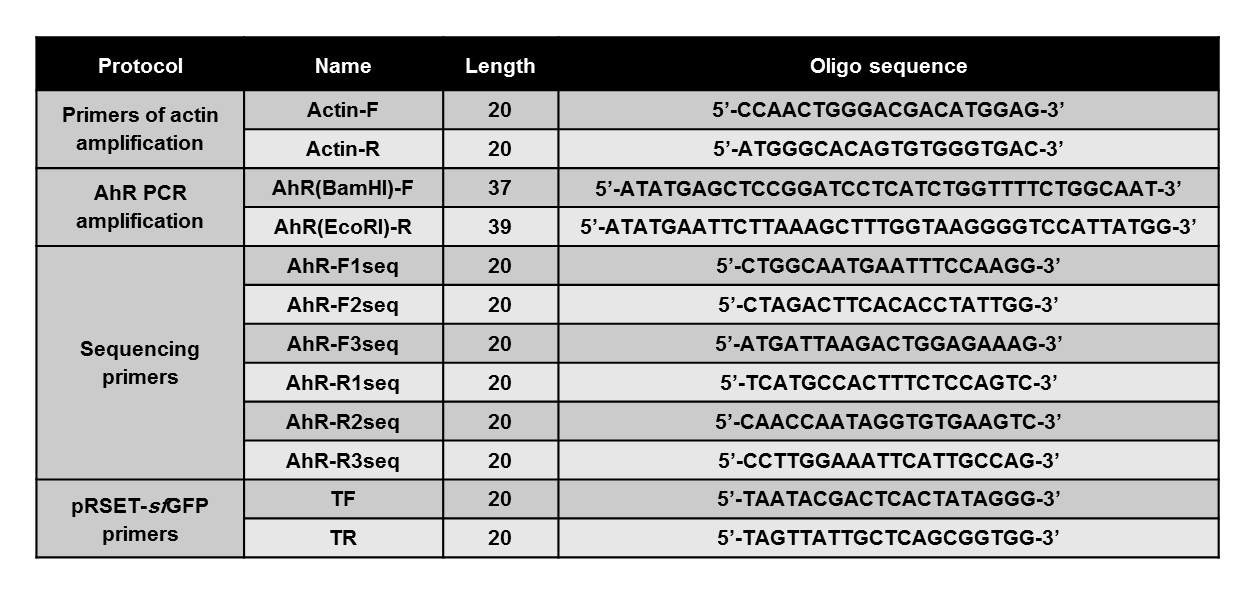

Supplement: Additional file 1: Table S1. — The primers used for the amplification and cloning of the AhR. The different parameters (application, name, length and sequence) of the primers used for AhR gene amplification and cloning into the pRSET-sfGFP plasmid. (DOC 79 kb) [file 12896_2016_282_MOESM1_ESM.doc]
