# Supplementary material for: Immuno-detection of dioxins using a recombinant protein of aryl hydrocarbon receptor (AhR) fused with sfGFP
Source: BMC Biotechnol. 2016 Jun 21;16:51. doi: 10.1186/s12896-016-0282-9 (PMC4915173; doi:10.1186/s12896-016-0282-9)

Additional file 2: Table S2 The different additives used for refolding the *sf*GFP-AhR. The basic information of the different additives used for solubilizing the *sf*GFP-AhR from the inclusion bodies.


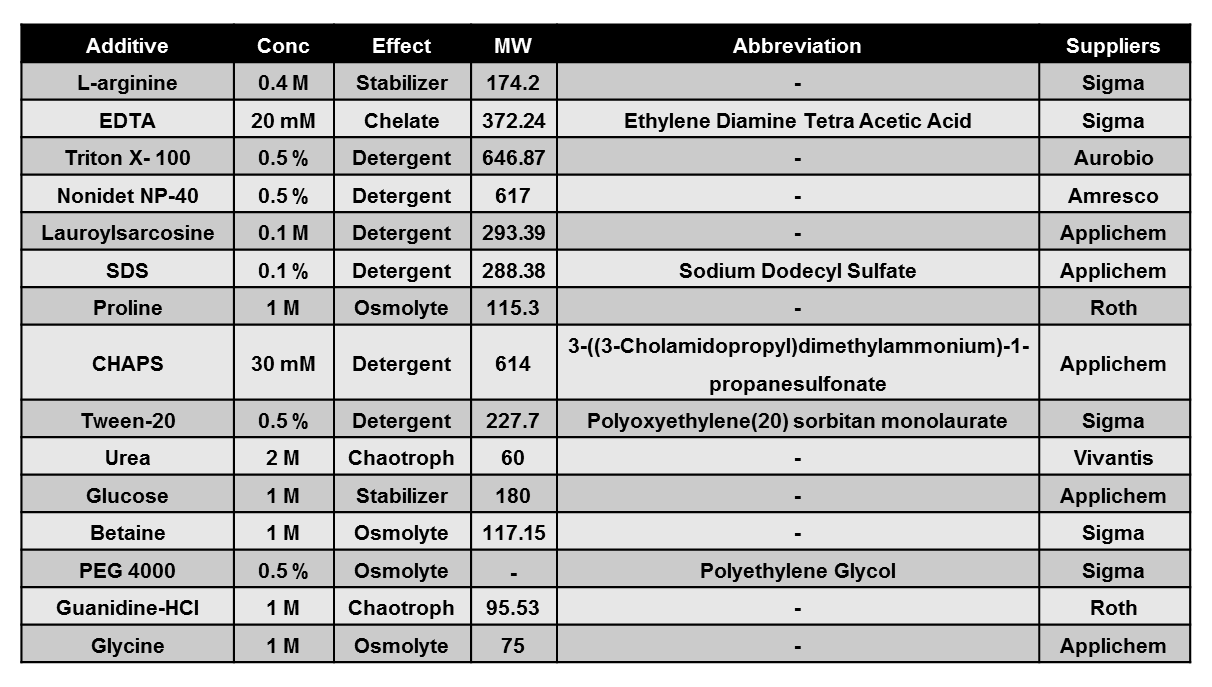

Supplement: Additional file 2: Table S2. — The different additives used for refolding the sfGFP-AhR. The basic information of the different additives used for solubilizing the sfGFP-AhR from the inclusion bodies. (DOC 95 kb) [file 12896_2016_282_MOESM2_ESM.doc]
