# Supplementary material for: Immuno-detection of dioxins using a recombinant protein of aryl hydrocarbon receptor (AhR) fused with sfGFP
Source: BMC Biotechnol. 2016 Jun 21;16:51. doi: 10.1186/s12896-016-0282-9 (PMC4915173; doi:10.1186/s12896-016-0282-9)
Supplement: Additional file 3: Figure S1. — Alignment of the AhR(LBD) amino acids sequences. Amino acids sequence alignment of the AhR(LBD) from human (hAhR, NP_001612), mouse (mAhR, NP_038492) and rat (rAhR, NP_037281). Similarity bar above the sequences and the different secondary structures (as attributed by Geneious software) are indicated with small arrows. The TCDD binding cavity and the PAS-B domains are indicated through which small boxes under the sequence refer to the amino acids whose side chains are implicated in the structure of the cavity (light coloured for lateral and dark for internal positions), as described before [31]. (DOC 272 kb) [file 12896_2016_282_MOESM3_ESM.doc]

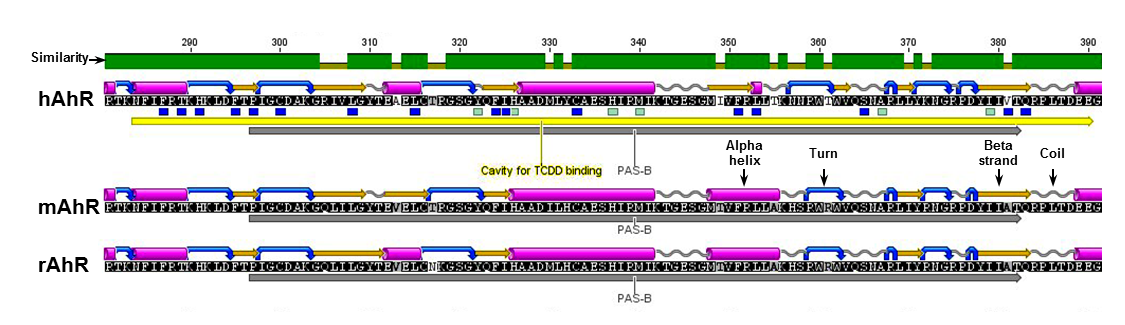


**Additional file 3: Fig. S1 Alignment of the AhR(LBD) amino acids sequences.** Amino acids sequence alignment of the AhR(LBD) from human (hAhR, NP_001612), mouse (mAhR, NP_038492) and rat (rAhR, NP_037281). Similarity bar above the sequences and the different secondary structures (as attributed by Geneious software) are indicated with small arrows. The TCDD binding cavity and the PAS-B domains are indicated through which small boxes under the sequence refer to the amino acids whose side chains are implicated in the structure (light coloured for lateral and dark for internal positions) of the cavity, according to .
