# Supplementary material for: Immuno-detection of dioxins using a recombinant protein of aryl hydrocarbon receptor (AhR) fused with sfGFP
Source: BMC Biotechnol. 2016 Jun 21;16:51. doi: 10.1186/s12896-016-0282-9 (PMC4915173; doi:10.1186/s12896-016-0282-9)
Supplement: Additional file 4: Figure S2. — The structure of the plasmid pRSET-sfGFP-AhR. Map of the plasmid construct pRSET-sfGFP-AhR, in which the inserted AhR (LBD) is indicated. The most important elements of the plasmid are shown, including the T7 promoter, N-terminal 6×His tag, two restriction sites (BamHI/EcoRI) used for insert ligation, ampicillin resistance gene (Amp), f1/PUC origin of replication and the sfGFP gene. (DOC 266 kb) [file 12896_2016_282_MOESM4_ESM.doc]

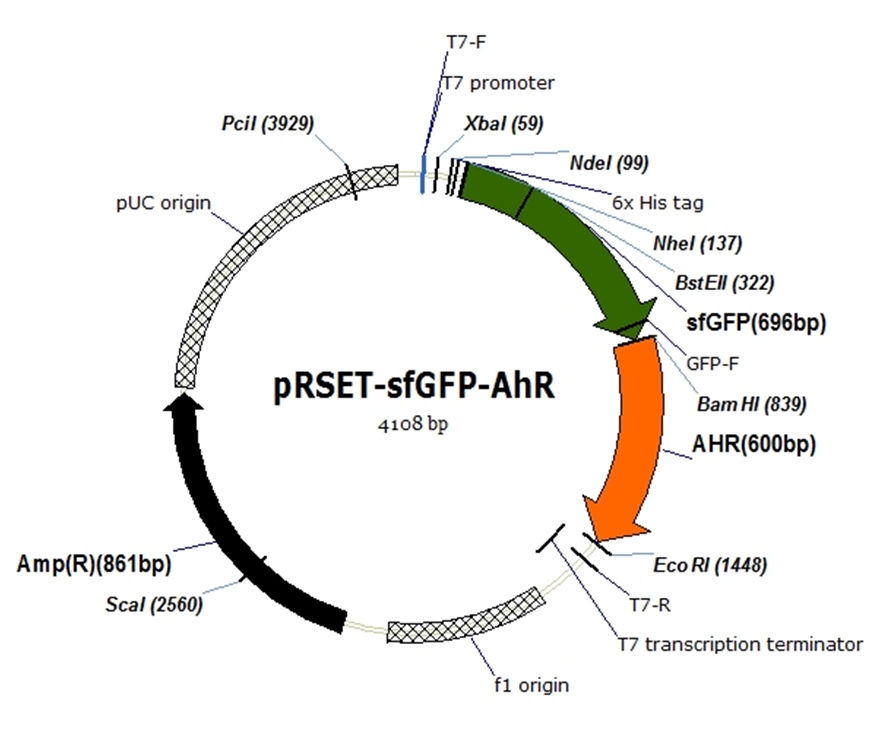


Additional file 4: Fig. S1 The structure of the plasmid pRSET-*sf*GFP-AhR. Map of the plasmid construct pRSET-*sf*GFP-AhR, in which the inserted AhR (LBD) is indicated. The most important elements of the plasmid are shown, including the T_7_ promoter, N-terminal 6×His tag, two restriction sites (*Bam*HI/*Eco*RI) used for insert ligation, ampicillin resistance gene (Amp), f1/PUC origin of replication and the *sf*GFP gene.
